# Neuronal Intrinsic Physiology Changes During Development of a Learned Behavior

**DOI:** 10.1523/ENEURO.0297-17.2017

**Published:** 2017-10-20

**Authors:** Matthew T. Ross, Diana Flores, Richard Bertram, Frank Johnson, Richard L. Hyson

**Affiliations:** 1 Program in Neuroscience, Florida State University, Tallahassee, FL 32306-4301; 2 Department of Psychology, Florida State University, Tallahassee, FL 32306-4301; 3 Department of Mathematics, Florida State University, Tallahassee, FL 32306-4501; 4 Program in Molecular Biophysics, Florida State University, Tallahassee, FL 32306-4380

**Keywords:** birdsong, HVC, intrinsic plasticity, ion channels, modeling, zebra finch

## Abstract

Juvenile male zebra finches learn their songs over distinct auditory and sensorimotor stages, the former requiring exposure to an adult tutor song pattern. The cortical premotor nucleus HVC (acronym is name) plays a necessary role in both learning stages, as well as the production of adult song. Consistent with neural network models where synaptic plasticity mediates developmental forms of learning, exposure to tutor song drives changes in the turnover, density, and morphology of HVC synapses during vocal development. A network’s output, however, is also influenced by the intrinsic properties (e.g., ion channels) of the component neurons, which could change over development. Here, we use patch clamp recordings to show cell-type-specific changes in the intrinsic physiology of HVC projection neurons as a function of vocal development. Developmental changes in HVC neurons that project to the basal ganglia include an increased voltage sag response to hyperpolarizing currents and an increased rebound depolarization following hyperpolarization. Developmental changes in HVC neurons that project to vocal-motor cortex include a decreased resting membrane potential and an increased spike amplitude. HVC interneurons, however, show a relatively stable range of intrinsic features across vocal development. We used mathematical models to deduce possible changes in ionic currents that underlie the physiological changes and to show that the magnitude of the observed changes could alter HVC circuit function. The results demonstrate developmental plasticity in the intrinsic physiology of HVC projection neurons and suggest that intrinsic plasticity may have a role in the process of song learning.

## Visual Abstract

**Figure:**
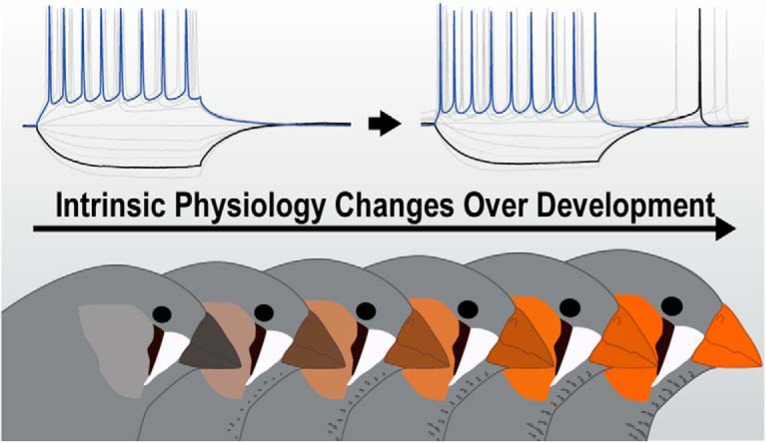

## Significance Statement

Models of learning commonly focus on changes in synaptic connectivity. Changes in the intrinsic properties of neurons (ion channels), however, may also produce changes in the function of neural circuits. The present experiments show that the intrinsic physiology of neurons in the cortical premotor nucleus HVC change over the course of song learning in the zebra finch. Consequently, models of song learning should account for these intrinsic changes along with changes in synaptic connectivity. More broadly, models of learning and memory should consider intrinsic plasticity of neurons as a possible contributor to how the nervous system encodes new information or novel behaviors.

## Introduction

Motor behaviors, such as speech, require the production of precise sequences of muscle activation (Simonyan and Horwitz, 2011). Consequently, the brain must encode accurate sequences of neuronal activity, which are determined by two components: the intrinsic physiology of the neurons in the circuit, and the connectivity between those neurons. Traditionally, studies of the physiology of learned behaviors have focused on synaptic changes and tacitly assume a stable intrinsic physiology. Indeed, many network models of learning focus solely on altering synaptic weighting between neurons to account for the encoding of novel behaviors (Fiete et al., 2010; Bernacchia, 2014; Sinha et al., 2014; Zheng and Triesch, 2014; Bennett and Bair, 2015; Ocker et al., 2015; Rajan et al., 2015). It remains possible, however, that the intrinsic properties of participating neurons (passive membrane properties and composition of ion channels) also change during learning. Indeed, changes in ion channels have been observed in a variety of systems and there is a growing appreciation of the possibility of nonsynaptic forms of plasticity contributing to the learning process (Debanne et al., 2003; Mozzachiodi and Byrne, 2010; Sehgal et al., 2013; Cansler et al., 2017). The present experiments explore changes in the intrinsic physiology of neurons in the zebra finch (*Taeniopygia guttata*) as juvenile birds progress through stages of vocal development.

Juvenile zebra finches learn their songs through a process of sensory-dependent imitation of an adult tutor (Brainard and Doupe, 2002). Vocal development occurs over distinct auditory and sensorimotor stages (Fig. 1; Tchernichovski et al., 2001). The auditory stage begins when a juvenile bird is exposed to the song of an adult tutor, resulting in the encoding of an auditory memory of the tutor song. The sensorimotor stage begins with the vocal production of subsong, which shows many acoustic similarities to the unpatterned babbling of human infants (Lipkind et al., 2013). Later, birds advance to plastic song where unpatterned vocalizations gradually differentiate into a facsimile of the syllable repertoire and sequence of the tutor song. Birds require auditory feedback throughout the sensorimotor stage to progressively shape vocal output toward the auditory memory of the tutor (Price, 1979).

**Figure:**
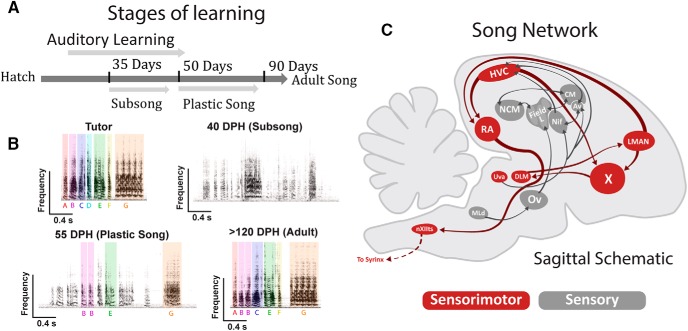
Figure 1. The developmental timeline and song circuit of zebra finches. ***A***, Standard timeline for song development by juvenile male zebra finches. The sensorimotor stage begins around 35 dph. By ∼90 dph, a male’s song is largely crystalized, remaining stable for the duration of adult life. ***B***, Example song spectrograms from experimental subjects. All birds were tutored to the same song pattern. ***C***, An abridged sagittal schematic of the song network in the male zebra finch. Note that HVC contains two major populations of projection neurons (HVC_X_ neurons and HVC_RA_ neurons) and interneurons (HVC_INT_). LMAN, lateral portion of the magnocellular nucleus of the anterior nidopallium; RA, robust nucleus of the arcopallium; DLM, medial dorsolateral nucleus of the thalamus; NCM, caudal medial nidopallium; NIF, forebrain nucleus interface of the nidopallium; CMM, caudal medial mesopallium; Av, nucleus avalanche; field L; Ov, ovoidalis; Uva, nucleus uvaeformis; MLd, dorsal lateral nucleus of the mesencephalon; nXIIIts, nucleus XII, tracheosyringeal part.

The present experiments focus on the cortical premotor nucleus HVC (Fig. 1), which is required for auditory learning (Roberts et al., 2010, 2012), for sensorimotor learning (Aronov et al., 2008), and for the production of the adult vocal pattern (Long and Fee, 2008). HVC’s key roles in the learning and production of song make it an exceptional target for exploring possible changes in intrinsic physiology of neurons related to behavioral development.

HVC contains three major populations of neurons: (1) HVC_RA_ neurons that project to the robust nucleus of the arcopallium (RA), the avian homolog of human laryngeal motor cortex; (2) HVC_X_ neurons that project to Area X (basal ganglia homolog), an area essential for song learning; and (3) interneurons (HVC_INT_) that synapse within the nucleus. Each cell type has a distinct physiology (Dutar et al., 1998; Kubota and Taniguchi, 1998; Daou et al., 2013). Our lab has previously developed conductance-based models of the three adult neuron types and pharmacologically tested the ion channel composition of the cells (Daou et al., 2013), setting the groundwork for exploring possible developmental changes in physiology during song learning.

Is it only the connections between HVC cells that are modified as the song is learned, or do the neurons themselves change? To answer this question, we obtained patch clamp recordings from HVC neurons during vocal development. This experimental study was supplemented by the development of conductance-based mathematical models of the neurons, to postulate how gradated changes in conductances may underlie the observed changes in neuronal physiology. We conclude that cell-type-specific developmental changes in the intrinsic properties of HVC projection neurons do in fact occur and that the magnitude of these changes is sufficient to alter the output of a neural circuit. Together, these findings suggest that plasticity of intrinsic physiology may play a role in the song learning process.

## Materials and Methods

### Experimental subjects

Male zebra finches (*n =* 22) were used in all experiments and were either hatched in aviaries or in individual breeding chambers at Florida State University. Animal care and experimental procedures were performed in accordance with National Science Foundation guidelines and approved by the Florida State University Animal Care and Use Committee. Juvenile finches were raised with one of two male-female pairs of adult finches in acoustically isolated, climate controlled recording chambers. The male tutor of the second pair was the offspring of the first pair and was tutored to that male’s song pattern. Thus, all juveniles used in experiments were tutored to the same song. Juveniles were either the offspring of the above pairs or transferred from the breeding aviaries at 7 d post-hatch (dph). Vocalizations were recorded across developmental stages to identify each juvenile’s stage of vocal development at the time of electrophysiological recording.

### Stages of vocal development

Electrophysiological recordings were made at three behavioral timepoints related to vocal development, categorized as subsong, plastic song, or adult song. Figure 1 shows the behavioral timeline and example vocalizations for finches used in these experiments along with a schematic detailing the circuitry of the song system.

#### Subsong

Subsong is the initial stage of song production during which vocalizations are largely unpatterned. Subsong typically begins at 30–35 dph. By 40 dph, all birds will be producing subsong. Importantly, housing with an adult male up to 40 dph is sufficient to establish an auditory memory of tutor song (Elliott et al., 2014). Birds in the subsong group (*n* = 5) were 39–42 dph.

#### Plastic song

The onset of plastic song is typically between 45–50 dph, when juveniles begin to produce syllables that resemble tutor syllables (Fig. 1). The transition from subsong to plastic song is marked by an increase in syllable complexity measured by increased variance in the pitch, pitch goodness, and entropy of syllables (Tchernichovski et al., 2001). Plastic song was objectively defined as a >70% increase in syllable spectral variance (a composite measure of pitch, pitch goodness, and entropy) when compared to subsong (Elliott et al., 2014). Birds in the plastic song group (*n* = 7) were 54–58 dph.

#### Adult song

Adult song is defined as a stable vocal pattern where there is minimal variability in syllable repertoire and sequence across bouts of singing. Finches typically sing a stable song pattern by 90 dph. Birds in the adult song group (*n* = 10) were >120 dph.

### HVC and cell identification using retrograde tracers

Viewed from a dorsal perspective, HVC is easily identifiable in adult finches based on its distinctive ovoid shape and heavy myelination (Daou et al., 2013). In juveniles, however, HVC is more difficult to distinguish due to reduced myelination (Herrmann and Bischof, 1986). To definitively identify HVC in juvenile birds and provide confirmation of specific cell types, a retrograde tracer was bilaterally injected into Area X, where it was taken up by HVC_X_ projection neurons and transported back to the cell bodies in HVC. Birds were deeply anesthetized with Equithesin and placed into a stereotaxic instrument. The scalp was resected, exposing the skull. Using the bifurcation of the midsagittal sinus to set the stereotaxic zero point and as a reference for head angle, craniotomies were made over Area X at 3.5 mm anterior to the stereotaxic zero and 1.5 mm lateral to the midsagittal sinus (Basista et al., 2014). The tracer DiI (Life Technologies; ∼400 nl volume) was bilaterally pressure injected 4 mm ventral from the surface of the brain into Area X via a glass micropipette using a gas pressure injection system (Applied Scientific Instrumentation MPPI-3). Dye was allowed to transport for at least 7 d before electrophysiological experiments. During patch clamp experiments HVC_X_ neurons were identified using epifluorescence illumination.

### Slice preparation

Subjects were anesthetized with isoflurane and rapidly decapitated. A section of the skull was blocked and then transferred to a dissecting chamber containing artificial cerebral spinal fluid (ACSF). The ACSF contained 119 mM NaCl, 2.5 mM KCl, 1.3 mM MgCl_2_, 2.5 mM CaCl_2_, 1 mM NaH_2_PO_4_, 26.2 mM NaHCO_3_, and 22 mM glucose. The osmolality of the ACSF was between 290–295 mOsm and the pH was ∼7.2. Throughout the procedure, all ACSF was gassed with 95% O_2_-5% CO_2_. After blocking, the brain was dissected out of the skull and the hippocampus was resected to aid in the sectioning of HVC. The hemispheres were separated and transferred to a vibrating microtome where parahorizontal sections of ∼250–300 µm were taken. The sections were transferred to an incubation chamber for approximately 1 h before the start of electrophysiological recording.

### Whole-cell electrophysiology

After tissue incubation, slices were transferred to a recording chamber containing perpetually oxygenated, room temperature ACSF. Recording electrodes were pulled to a resistance of 5–9 MΩ using a Sutter Instruments P-80 micropipette puller and filled with intracellular solution using hand pulled injectors. Electrodes were unpolished. The intracellular solution contained the following: 125 mM K-gluconate, 15 mM KCl, 1 mM MgCl_2_, 10 mM HEPES, 5 mM EGTA, 2 mM Mg-ATP, and 0.3 mM Na_3_-GTP, pH was adjusted to ∼7.2 using KOH and the osmolality was adjusted to ∼295–300 mOsm using sucrose. The AMPA/kainate receptor antagonist CNQX and the GABA_A_ receptor antagonist picrotoxin were bath applied to limit synaptic input to the neurons. Neurons were visualized under a 40× water immersion objective using a camera system attached to an Olympus BX51 microscope. Epifluorescence illumination was used to identify HVC and identify HVC_X_ neurons. Electrodes were guided to cells using IR-DIC illumination. Positive pressure was applied to the electrode before being visually guided to a neuron. After observing a dimple in the cell membrane and an increase in pipette resistance, positive pressure was removed and a small amount of negative pressure was mouth applied through the electrode holder. Once a gigaohm seal was attained, short pulses of negative pressure were mouth applied until the seal was ruptured. Provided that the neuron showed a stable resting potential below approximately −50 mV, a stable access resistance and exhibited action potentials in response to depolarizing current injection, it would undergo a battery of current injection protocols detailed below. The liquid junction potential was measured to be approximately −2.8 mV and was left uncorrected.

### Electrophysiological protocols and feature analysis

Recordings were made using a Multiclamp 700B amplifier connected to a computer running pClamp 9 or 10 (Molecular Devices). For most neurons, whole-cell current clamp was used to first determine the passive membrane properties of the neurons by repeatedly injecting a 100 ms pulse of −10 pA of current over 10 sweeps. This small hyperpolarizing current minimizes the activation of voltage-dependent ion channels (Franzen et al., 2015; Hong et al., 2016). The sweeps were then averaged. The membrane input resistance (R_M_) was calculated by dividing the magnitude of the voltage deflection during the current injection by the value of the applied current (−10 pA). The membrane time constant (τ_M_) was calculated using a single exponential fit to the first 50 ms after current onset. The membrane capacitance (C_M_) was then calculated by dividing the membrane time constant by the membrane input resistance (C_M_ = τ_M_/R_M_). Square current pulses of 300 ms duration were then applied and the voltage responses were analyzed and compared across neurons for a variety of features. All analyses were done using custom MATLAB analysis scripts using the calculations detailed below.

**Figure 2.**
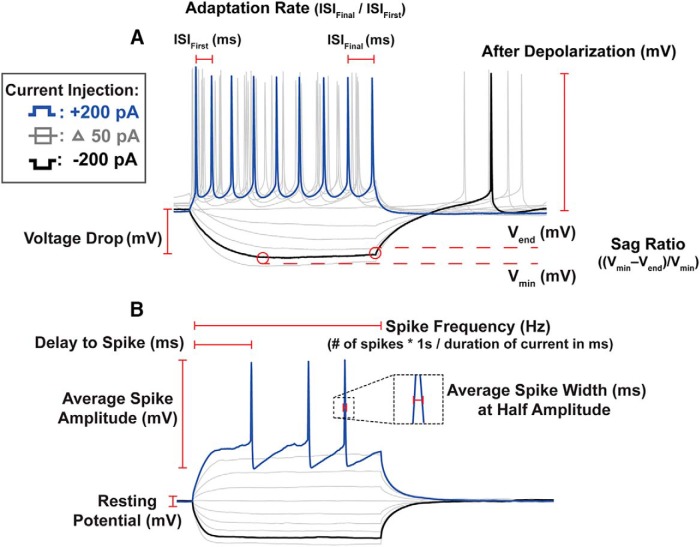
Electrophysiolgical trace analysis. ***A***, Example from an adult HVC_X_ neuron. ***B***, Example from an adult HVC_RA_ neuron. All traces were analyzed using an automated script that measured a variety of features for each voltage trace. See Materials and Methods for a full list of the features analyzed, along with a detailed explanation of measurment procedures. Many of the depolarizing features (e.g., spike ampltide, adaptation rate) were analyzed at a current injection of 200 pA (blue traces). Many hyperpolarizing features (e.g., SR, rebound depolarization) were analyzed at −200 pA (black traces).

Figure 2 illustrates many of the measured features. Neurons were stimulated for a duration of 300 ms. The applied current typically ranged from at least −200 to 200 pA. The resting membrane potential (V_rest_) was calculated by averaging the voltage of the trace before the onset of the current injection. Average spike amplitude was defined as the mean of the first spike amplitude for all depolarizing current injections, calculated as the maximum voltage minus the minimum voltage of the after-hyperpolarization of the spike. Average spike-width was defined as the mean of the spike-width calculated at half amplitude for the first spike of all depolarizing current injections. Spike latency was calculated as the time required to elicit a spike after the onset of a 200 pA current injection. Spike frequency was calculated by multiplying the number of spikes elicited by a 200 pA current injection over the current pulse by 1000/duration of pulse to obtain spikes/sec. This gives the mean spike frequency of the neuron over the duration of the current pulse. Inter-spike interval was calculated as the time in milliseconds between spikes at half-amplitude. Spike frequency adaptation was calculated by computing the adaptation ratio (AR) between the final inter-spike interval and the first inter-spike interval for pulses of 200 pA. An AR of 1 indicates there is no adaptation whereas a larger AR indicates increased a slowdown of spiking. The sag ratio (SR) was calculated as (V_min_ – V_end_)/V_min_ such that V_min_ is the minimum voltage during the hyperpolarizing pulse of −200 pA, and V_end_ is the final voltage at the offset of the current injection. The voltage drop (V_drop_) during hyperpolarization was measured as V_rest_ – V_min_ for a hyperpolarizing current of −200 pA.

### Experimental design and statistical analysis

A total of *n* = 5 birds were recorded at subsong, *n* = 7 birds at plastic song and *n* = 10 birds at adult song. The number of cells recorded at each age group are detailed in Results. To compare across age groups the values for each measured feature were averaged together and compared using a one-way ANOVA with three levels of comparison (each age group) followed by *post hoc* test; *p* values (Table 1) were adjusted for multiple comparisons using a Bonferroni correction. This method, common to many statistical packages, multiplies the uncorrected *p* value by the number of comparisons (in our case three) and is functionally equivalent to applying the correction to the alpha value. If the resulting, corrected, *p* > 1, it is rounded down to 1. Group means were considered to be statistically different if the *post hoc* test reached *p* < 0.05. Statistics were computed with MATLAB using the ANOVA and multcompare functions. All data points are shown for the features that showed major developmental changes or were indicative of a cellular phenotype.

**Table 1.** Experimental results of features measured for each cell type

HVC_X_	Subsong	Plastic song	Adult	Subsong vs plastic	Subsong vs adult	Plastic vs adult
Resting potential (mV)	−67 ± 1.51	−68 ± 1.54	−67 ± 1.50	*p* = 1.00	*p* = 1.00	*p* = 1.00
Membrane_Time Constant_ (ms)	33 ± 4.28	39 ± 7.10	43 ± 7.13	*p* = 1.00	*p* = 0.84	*p* = 1.00
Membrane_Input Resistance_ (MΩ)	282 ± 52.65	115 ± 22.72	296 ± 39.11	*p* = 0.04	*p* = 1.00	*p* = 0.01
Membrane_Capacitance_ (pF)	156 ± 38.95	308 ± 38.54	167 ± 28.68	*p* = 0.03	*p* = 1.00	*p* = 0.03
V_drop_ (mV)	30 ± 2.72	26 ± 2.31	46 ± 5.37	*p* = 1.00	*p* = 0.02	*p* = 0.00
SR	0.01 ± 0.0013	0.02 ± 0.0046	0.04 ± 0.0065	*p* = 0.17	*p* = 0.00	*p* = 0.03
Rebound depolarization (mV)	1.7 ± 0.39	3.3 ± 0.52	40.5 ± 11.10	*p* = 1.00	*p* = 0.00	*p* = 0.00
Spike frequency (Hz)	27 ± 4.61	19 ± 2.31	28 ± 2.65	*p* = 0.26	*p* = 1.00	*p* = 0.25
Adaptation rate	1.55 ± 0.11	1.79 ± 0.21	1.75 ± 0.13	*p* = 0.82	*p* = 1.00	*p* = 1.00
Spike amplitude (mV)	65 ± 4.98	82 ± 5.61	84 ± 4.17	*p* = 0.06	*p* = 0.03	*p* = 1.00
Spike width (ms)	2.19 ± 0.14	1.56 ± 0.15	1.87 ± 0.16	*p* = 0.02	*p* = 0.45	*p* = 0.46
Spike latency (ms)	56 ± 23.69	36 ± 9.46	19 ± 3.90	*p* = 1.00	*p* = 0.33	*p* = 1.00
HVC_RA_						
Resting potential (mV)	−61 ± 2.05	−66 ± 0.94	−77 ± 0.57	*p* = 0.01	*p* = 0.00	*p* = 0.00
Membrane_Time Constant_ (ms)	28 ± 4.09	17 ± 2.48	18 ± 2.53	*p* = 0.10	*p* = 0.11	*p* = 1.00
Membrane_Input Resistance_ (MΩ)	364 ± 36.04	339 ± 55.66	327 ± 51.93	*p* = 1.00	*p* = 1.00	*p* = 1.00
Membrane_Capacitance_ (pF)	83 ± 21.28	66 ± 22.17	68 ± 12.24	*p* = 1.00	*p* = 1.00	*p* = 1.00
V_drop_ (mV)	30 ± 6.55	29 ± 6.01	28 ± 3.80	*p* = 1.00	*p* = 1.00	*p* = 1.00
SR	0.01 ± 0.0041	0.02 ± 0.0036	0.01 ± 0.0009	*p* = 0.18	*p* = 1.00	*p* = 0.01
Rebound depolarization (mV)	1.2 ± 0.60	1.8 ± 0.55	1.1 ± 0.20	*p* = 1.00	*p* = 1.00	*p* = 0.76
Spike frequency (Hz)	10 ± 5.12	10 ± 3.84	7 ± 2.12	*p* = 1.00	*p* = 1.00	*p* = 1.00
Adaptation rate	2.05 ± 0.40	1.01 ± 0.28	1.34 ± 0.18	*p* = 0.09	*p* = 0.27	*p* = 1.00
Spike amplitude (mV)	50 ± 6.39	64 ± 3.29	77 ± 3.59	*p* = 0.16	*p* = 0.00	*p* = 0.05
Spike width (ms)	1.94 ± 0.18	1.75 ± 0.29	1.82 ± 0.17	*p* = 1.00	*p* = 1.00	*p* = 1.00
Spike latency (ms)	15 ± 8.07	92 ± 40.53	17 ± 5.72	*p* = 0.05	*p* = 1.00	*p* = 0.02
HVC_INT_						
Resting potential (mV)	−60 ± 5.87	−58 ± 1.68	−57 ± 1.44	*p* = 1.00	*p* = 1.00	*p* = 1.00
V_drop_ (mV)	40 ± 6.46	37 ± 3.21	47 ± 6.34	*p* = 1.00	*p* = 1.00	*p* = 0.51
SR	0.13 ± 0.0267	0.11 ± 0.0190	0.13 ± 0.0204	*p* = 1.00	*p* = 1.00	*p* = 1.00
Rebound depolarization (mV)	68.3 ± 5.52	61.6 ± 9.25	56.0 ± 2.87	*p* = 1.00	*p* = 0.85	*p* = 1.00
Spike frequency (Hz)	79 ± 18.06	77 ± 14.18	105 ± 13.55	*p* = 1.00	*p* = 0.84	*p* = 0.51
Adaptation rate	1.20 ± 0.08	1.44 ± 0.18	1.18 ± 0.07	*p* = 0.83	*p* = 1.00	*p* = 0.49
Spike amplitude (mV)	67 ± 6.00	64 ± 3.02	58 ± 2.17	*p* = 1.00	*p* = 0.25	*p* = 0.53
Spike width (ms)	0.85 ± 0.13	1.16 ± 0.15	0.98 ± 0.09	*p* = 0.38	*p* = 1.00	*p* = 0.91
Spike latency (ms)	6 ± 2.86	5 ± 1.22	4 ± 0.00	*p* = 1.00	*p* = 1.00	*p* = 1.00

The means, SEMs, and significance values (Bonferroni-corrected pairwise comparisons) for all features mesured of each cell type across each age group.

### Mathematical modeling

Single-compartment conductance-based neuron models were used to predict changes in ion channel properties that may underlie the changes in features of the voltage response to applied currents that occur across development. Modeling is necessary to describe our experimental results as the models can predict gradated changes in ion channel conductance. Our approach was to fit model parameters to a representative neuron from each subpopulation (i.e., juvenile or adult), and then compare the values to determine which conductances changed substantially. This approach is preferable to fitting models to population averages, since an “average neuron” may, in fact, not behave like any of the neurons in the population (Golowasch et al., 2002). An alternative averaging approach, fitting parameters to all cells within a subpopulation and then averaging those values, would be similarly problematic.

Our models build on the work of Daou et al. (2013), where models were developed and tested using pharmacology to confirm the presence and the role of several ion channels in shaping the intrinsic physiology of adult HVC neurons. Using the Daou et al. (2013) models as a foundation, we used fits to our developmental data to predict gradated variations in channel properties across development. This approach, of course, assumes that the conductances we previously determined to be present in adult neurons include all of the critical conductances present in juvenile neurons. That is, no juvenile-only conductance is responsible for age-dependent differences in physiology. This alternative seems unlikely since good fits to physiologic data were obtained using models based solely on a common set of conductances. The components of this model are briefly described below, and the source code for the original model (Daou et al., 2013) and the models developed in this report are available at http://www.math.fsu.edu/∼bertram/software/birdsong. The code is available as Extended Data 1.

**Figure 3.**
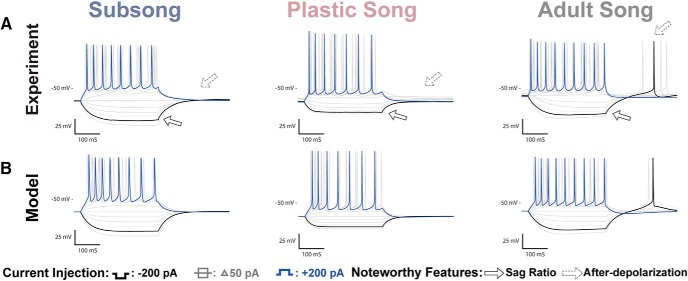
HVC_X_ recordings and coresponding model traces. ***A***, Voltage traces of HVC_X_ neurons recorded at subsong, plastic song, or adult stages of development. Features that showed developmental changes included an increase in the Sag ratio (solid arrow) and an increase in rebound depolarization that resulted in rebound spiking (dotted arrow). ***B***, Model traces of the three neurons depicted in ***A***. The models attribute the sag to the h-current and rebound firing to the T-type Ca^2+^ current.

10.1523/ENEURO.0297-17.2017.ed1 Extended Data 1 Matlab code (.m files) for biophysical models of HVC neurons depicted in Figures 3 and 5. XPPAUT code (.ode files) for network models depicted in Figure 9. Download Extended Data 1, ZIP file.

The models consist of voltage-gated Na^+^ and K^+^ currents (*I_Na_* and *I_K_*), a hyperpolarization-activated cation current (*I_h_*), a high-threshold L-type Ca^2+^ current (*I_Ca-L_*), a low threshold T-type Ca^2+^ current, a small conductance Ca^2+^-activated K^+^ current (*I_SK_*), a persistent Na^+^ current (*I_Nap_*), an M-type K^+^ current (*I_M_*), an A-type K^+^ current (*I_A_*), and a leak current (*I_L_*). Different values for capacitance and other parameters are used for the different neuron types and different stages of development. The currents were modeled as in Daou et al. (2013) with two exceptions. First, the M-current replaced the Na^+^ dependent K^+^ current (I_KNa_) from Daou et al. (2013), as the pharmacological evidence for I_KNa_ was equivocal given the nonselectivity of blockers. Additionally, modeling the M-current requires fewer assumptions than I_KNA_ since it can be treated as a strictly voltage-dependent current, rather than a current that is dependent on hypothetical changes in intracellular ion concentrations. Second, the present models updated the kinetics of the h-current used by Daou et al. (2013) so as to match experimental data from zebra finch neurons in the dorsolateral nucleus of the thalamus (Luo and Perkel, 2002). Both of these changes improved the fit of our models to the experimental data.

The membrane potential is determined by the following equation:

$$C\frac{dV}{dt}=-(I_{L}+I_{K}+I_{Na}+I_{Nap}+I_{CaL}+I_{CaT}+I_{A}+I_{SK}+I_{M}+I_{h}-I_{App})$$

where C is the cell membrane capacitance, and *I_App_* is the applied current.

The updated kinetics equation for the h-current is

$$\overset{˙}{x}=\frac{h_{\infty }-x}{\tau_{x}},$$

where *x* = *s* or *f* represent the slow and fast activation variables of the h-current, respectively. The activation function is given by

$$h_{\infty }=\frac{1}{1+\exp\left(\frac{V+87.7}{6.4}\right)}$$

and the time constants by

$$\tau_{s}=exp\left(\frac{V+289.7}{33.3}\right)$$

and

$$\tau_{f}=\frac{100}{\frac{-7.4\left(V+70\right)}{\exp\left(\frac{V+70}{-0.8}\right)-1}+65exp\left(\frac{V+56}{-23}\right)}$$

The h-current is then

$$I_{h}=g_{h}(w_{h}f+\left(1-w_{h}\right)s)(V-V_{h})$$

where *w_h_* is the fraction of the h-conductance that has fast activation.

The M-current is modeled as in Golomb et al. (2006),

$$I_{M}=g_{M}z\left(V-V_{K}\right)$$

The kinetic equation and activation function are

$$\overset{˙}{z}=\frac{z_{\infty }-z}{\tau_{z}}$$

and

$$z_{\infty }=\frac{1}{1+\exp\left(\frac{V+\theta_{z}}{-5}\right)}.$$

Computer simulations of the models were performed using the MATLAB solver ode23.

A simple HVC circuit model was constructed to test how changes in intrinsic physiology could affect the output timing of HVC. The individual neuronal models consisted of the equations detailed above. The synaptic currents for the circuit model are described as in Destexhe et al. (1994) where transmitter release is given by

$$\left[Tr\left(V_{pre}\right)\right]=\frac{0.0015}{1+\exp\left(\frac{V_{pre}+5}{-4}\right)},$$

where *V_pre_* is the voltage of the presynaptic neuron, and the postsynaptic current’s kinetics depend on the bound fraction of postsynaptic receptors, given by

$$\dot{x}=\alpha_{x}\left(1-x\right)\left[Tr\left(V_{pre}\right)\right]-\beta_{x}x$$

The postsynaptic currents are

$$I_{syn}=g_{x}x(V_{post}-V_{syn}),$$

where *x* = *GABA* or *AMPA*, representing the activation variables for the inhibitory and excitatory currents, respectively, *V_post_* is the voltage of the postsynaptic neuron, and *V_syn_* is the corresponding reversal potential for GABA and AMPA channels. For inhibitory currents, $\alpha_{GABA}=5000\;M^{-1}{ms}^{-1}$, βGABA=0.18ms-1,gGABA=180nS,and Vsyn=-90mV. Excitatory currents have values of $\alpha_{AMPA}=1100M^{-1}{ms}^{-1}$, $\beta_{AMPA}=0.19{ms}^{-1},g_{AMPA}=20nS,and\;V_{syn}=0mV$. Circuit simulations were performed using XPPAUT (http://www.math.pitt.edu/∼bard/xpp/xpp.html).

## Results

The patch clamp recordings from adult subjects replicated previous work showing three distinct physiologic phenotypes of HVC neurons (Dutar et al., 1998; Kubota and Taniguchi, 1998; Daou et al., 2013). HVC_X_ neurons show spike-frequency adaptation, moderate inward rectification and rebound spiking. HVC_RA_ cells often fire only a few spikes, have a resting potential near −80 mV, and show no sag response to hyperpolarizing currents. HVC_INT_ neurons fire spontaneously, have high firing frequency compared to the other cell types and a large sag response to hyperpolarizing currents. HVC_X_ neurons were identified by the presence of the fluorescent tracer DiI. HVC_RA_ and HVC_INT_ neurons were identified by the lack of tracer and by the similarity of their responses to the established adult physiologic phenotype for that cell type, and their identification is referred to as “putative”. Recently, HVC to Nucleus Avalanche neurons have been identified as a distinct population (Roberts et al., 2017). Proportionally, these represent a small percentage of HVC projection neurons (≤1%) and possible developmental changes in their physiology were not explored in these experiments.

Patch clamp recordings showed that the intrinsic physiology of identified HVC_X_ neurons and putative HVC_RA_ neurons changed as a function of song development. Some physiologic features remained stable in one cell type but changed in another, indicating that there are cell-type-specific changes. The traces were analyzed for a variety of features and tested for statistically significant differences across age groups (see Materials and Methods). While only selected properties of interest are discussed in detail below, the population data and statistics for all analyzed features are shown in Table 1. Recordings from putative HVC_INT_ neurons showed that, as a population, their intrinsic properties were relatively stable across development.

Mathematical models were used to interpret the observed changes in physiologic features of projection neurons and identify possible changes in the ion channel expression patterns that underlie the developmental changes in intrinsic physiology. To validate the models, the same feature analysis performed on the experimental traces (Fig. 2) was applied to modeled outputs that were generated using applied currents with the same amplitude and duration as those used during the experimental recordings. Modeled outputs showed feature values within the range of experimentally recorded values for each physiologic feature.

### Developmental changes in HVCX neurons

**Figure 4.**
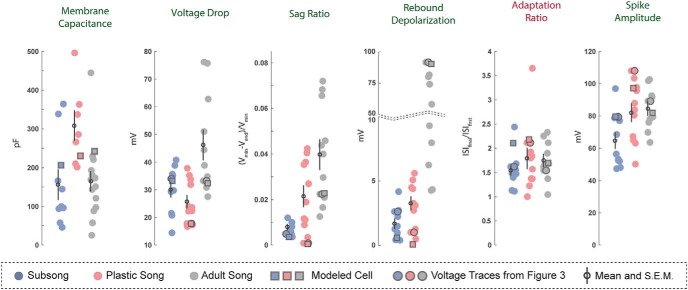
Developmental changes in HVCx neurons. Each scatterplot depicts a physiologic feature of interest where each point represents measurement from a single neuron. Outlined circles represent features measured from the experimentaly recorded traces presented in Figure 3. Outlined squares indicate the measurments from the modeled traces presented in Figure 3. The means and SEMs are ploted over each data set. Legends for developmental changes that were statistically significant are indicated in green font while features that were developmentally stable are indicated in red.

Examples of the physiologic responses of HVC_X_ neurons are shown in Figure 3, along with model simulations with parameters adjusted to fit the experimental traces. A total of *n* = 33 HVC_X_ neurons were recorded across development: *n =* 11 at subsong, *n =* 11 at plastic song, and *n =* 11 at adult song. Figure 4 shows all data points for some of the key features that were analyzed. Several developmental changes in the properties of HVC_X_ neurons reached statistical significance. There was a shift in the passive membrane input resistance and the membrane capacitance. In response to a hyperpolarizing applied current, there was an increased V_drop_ and SR, and an increased rebound depolarization following removal of the current. In response to a depolarizing current there was an increase in the spike frequency and spike amplitude and a decreased spike width (Table 1; Fig. 4, green headings). The specific details of physiologic changes are described below.

#### Passive membrane properties

Some passive membrane properties of HVC_X_ neurons varied during development. Although HVC_X_ neurons at all developmental timepoints had a similar resting potential and membrane time constant, membrane input resistance and capacitance showed a nonmonotonic developmental progression. The membrane input resistance was an average of 282 ± 52.65 MΩ at subsong, decreasing to 115 ± 22.72 MΩ at plastic song, and then increasing to 296 ± 39.11 MΩ at adult song. The average membrane capacitance was 156 ± 38.95 pF at subsong, increasing to 308 ± 38.54 pF at plastic song and returning to 167 ± 28.68 pF by adult song (Fig. 4; Table 1).

#### Response to hyperpolarizing applied current

Figure 3 shows sample responses of neurons at each developmental stage. For quantitative comparisons, responses to −200 pA current injections were analyzed when measuring the hyperpolarizing response of the cell. Adult HVC_X_ neurons showed a V_drop_ of 46 ± 5.37 mV and exhibited a sag response to hyperpolarizing currents (0.04 ± 0.0065 mV) that is often followed by a rebound spike or prominent after-depolarization on termination of the hyperpolarization. HVC_X_ neurons from subsong and plastic song showed a smaller V_drop_ of 30 ± 2.72 mV for subsong neurons and 26 ± 2.31 mV for plastic song neurons. Subsong HVC_X_ neurons showed little or no sag response, with an average SR of 0.01 ± 0.0013. The sag response was more variable among plastic song HVC_X_ neurons, with SR ranging from 0.00 to 0.04, with an average of 0.02 ± 0.0046. Some plastic song HVC_X_ neurons showed no sag, similar to subsong HVC_X_ neurons, while some had a SR comparable to that seen in adults.

Many adult HVC_X_ neurons exhibited rebound spiking. Those that did not had an after-depolarization averaging 7.0 ± 0.78 mV. In contrast, subsong HVC_X_ neurons never exhibited rebound spiking, having a limited after-depolarization of 1.7 ± 0.39 mV. Rebound firing was also not observed in plastic song HVC_X_ neurons, but they did show a moderate mean after-depolarization of 3.3 ± 0.52 mV.

#### Response to depolarizing applied current

HVC_X_ neurons at all developmental timepoints generated tonic spiking to depolarizing current injections. They showed an increased firing frequency at adult song (32 ± 1.72 Hz) when compared to subsong (24 ± 3.93 Hz) and plastic song (19 ± 2.31 Hz). All HVC_X_ neurons exhibited spike frequency adaptation during the 300 ms depolarizations. Although neurons at adult song showed a higher frequency of action potentials to the standard +200 pA current injection, cells at all three behavioral timepoints showed similar adaptation rates. Analyses of individual action potentials showed that, as a population, subsong HVC_X_ neurons had wider action potentials of shorter amplitude than neurons recorded at plastic or adult song; however, there was considerable overlap in the data across all developmental timepoints.

Figure 4 shows quantification of six of the features of the voltage traces at each developmental stage. Each data point reflects a feature from one neuron in response to a current pulse of ±200 pA, with the exception of the first panel, which shows the membrane capacitance. The large outlined circles indicate the neurons shown in Figure 3, while the small open circles are the mean of the sub-population. Features that changed significantly over development are indicated with a green heading, while those that did not are indicated with a red heading. We interpret the changes next, employing biophysical models to suggest how ion channel parameters change over development.

#### Mathematical models suggest developmental changes in channel parameters of HVC_X_ neurons

Each of the three model calibrations was made to a single neuron (Figs. 3*A*, 4, outlined circles). The feature quantification from the model traces are shown as outlined squares in Figure 4.

Model parameters for the two different juvenile timepoints were compared against parameter values for adult neurons (Fig. 7*A*, positive values indicate that the juvenile parameter value is greater than that for the adult). Only select parameter values that differed across age groups are listed in Figure 7; however, all parameter values can be found in Table 2.

**Table 2.** Model parameter values

Model parameter	Subsong HVC_RA_	Plastic song HVC_RA_	Adult song HVC_RA_	Subsong HVC_X_	Plastic song HVC_X_	Adult song HVC_X_
V_L_ (mV)	−62	−62	−77	−72	−75	−63
V_H_ (mV)	−43	−43	−43	−43	−43	−43
g_L_ (nS)	4	7	7	5.6	5	5
g_CaL_ (nS)	1	1	1	1	1	1
g_Na_ (nS)	560	440	300	1500	3100	2300
g_K_ (nS)	80	80	500	160	180	120
g_A_ (nS)	5	5	5	5	5	5
g_M_ (nS)	160	100	32	11.8	53	15.4
g_CaT_ (nS)	0.1	1	1	2	2	3.8
g_SK_ (nS)	1	5	32	0.4	2.8	2.1
g_h_ (nS)	0.8	2.2	1.6	0.4	4.1	2.25
*w_*h*_*	0.95	0.95	0.95	0.3	0.99	0.17
C (pF)	60	55	58	220	245	260
τ_h_ (ms)	1.2/(α_*h*_ + β_*h*_)	1.2/(α_*h*_ + β_*h*_)	1.2/(α_*h*_ + β_*h*_)	1.2/(α_*h*_ + β_*h*_)	1.2/(α_*h*_ + β_*h*_)	1.2/(α_*h*_ + β_*h*_)
σ_m_ (mV)	-5	-5	-10	-5	-8	-4
θ_m_ (mV)	-30	-32	-35	-36	-35	-38
$\overset{-}{\tau_{n}}$ (ms)	15	8	15	10	10	10
τ_z_ (ms)	75	75	75	26.25	45	75
θ_z_ (mV)	−29	−30	−45	−35	−39	−39
θ_n_ (mV)	−32	−30	−30	−30	−30	−30
σ_n_ (mV)	−5	−5	−7	−10	−7	−6
σ_s_ (mV)	−8.6	−8.6	−8.6	−8.6	−8.6	−8.6
θ_s_ (mV)	−13	−13	−13	−13	−13	−13
*f*	0.01	0.01	0.01	0.01	0.01	0.01
*k*_s_ (μM)	0.4	0.4	0.4	0.4	0.4	0.4
b_Ca_ (μM)	0.05	0.05	0.05	0.05	0.05	0.05
θ_aT_ (mV)	−59	−59	−59	−59	−59	−59
σ_aT_ (mV)	−6	−6	−6	−6	−6	−6

**Figure 5.**
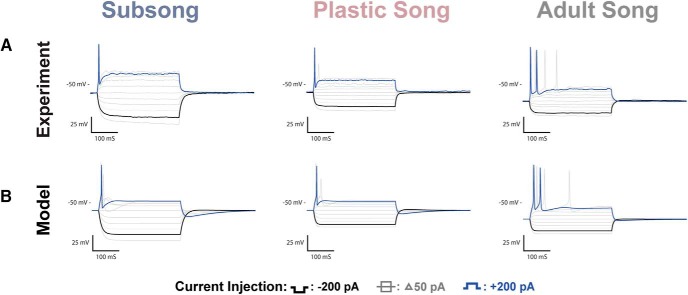
HVC_RA_ recordings and coresponding model traces. ***A***, Voltage traces of HVC_RA_ neurons recorded at subsong, plastic song, or adult stages of development. One of the more prominent changes observed across development was a systematic decrease in the resting potential of HVC_RA_ neurons. ***B***, Modeled traces of the three neurons depicted in ***A***. The models attributed the shift in resting potential to the leak current.

The parameter values for the model traces presented in Figures 3, 5.

HVC_X_ neurons showed three main developmental changes in their response to hyperpolarizing currents. The first significant feature change shown in Figure 4 is an increased V_drop_ in the adult HVC_X_ neurons compared with the juvenile. The model attributes this to a developmental decrease in the fast component of the h-current, relative to the slow component. This is reflected in the *w_h_* parameter (fraction of the h-current conductance that has fast activation), which is larger in the model cells of the juveniles than in the adults (Fig. 7*A*; Table 2). Thus, in the juveniles, hyperpolarization quickly activates a substantial h-current that opposes the hyperpolarizing current, limiting the V_drop_. In the adult, there is less of this current, so the V_drop_ is greater. The second significant feature change is a developmental increase in the SR. According to the model fitting, this is due to an increase in the slow component of the h-current, relative to the fast component, again reflected in the larger value of the *w_h_* parameter in the juveniles than in the adults. This slowly-activated component slowly depolarizes the membrane, resulting in a sag. The third significant feature change is an increase in the rebound depolarization, reflecting an increase in the number of neurons that spike following removal of hyperpolarizing current. The model fitting suggests that this is due to an increase in the conductance of the T-type Ca^2+^ current (*g_Ca-T_* is larger in the adult model cell than in the juveniles), as well as an increase in the slow component of the h-current. We showed previously that these currents are responsible for the rebound in HVC_X_ neurons (Daou et al., 2013).

One feature change in response to depolarizing currents was an increase in the spike frequency from plastic to adult cells. The model fittings suggest that this is due to a developmental decrease in the conductance and an increase in the time constant for activation of the M-type K^+^ channels (*g_M_*, τ_*z*_; Fig. 7*A*). In juveniles, this channel activates more rapidly, turning on more of the hyperpolarizing current during the depolarizing pulse. The result is a lower spike frequency in juvenile neurons. The effect of the SK current in these cells is small, as was shown in adult neurons in Daou et al. (2013). There were developmental variations in spike amplitude and spike width due to changes in the conductance of Na^+^ and delayed rectifying K^+^ currents (Table 2).

### Developmental changes in HVC_RA_ neurons

**Figure 6.**
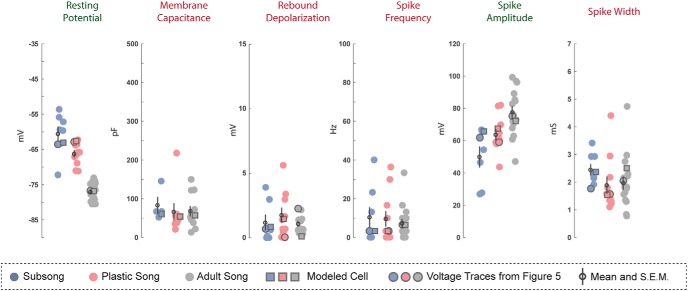
Developmental changes in HVC_RA_ neurons. Each scatterplot depicts a physiologic feature of interest where each point represents the measurement from a single neuron. Outlined circles represent features measured from the experimentaly recorded traces presented in Figure 5. Outlined squares indicate the measurments from the modeled traces presented in Figure 5. The means and SEMs are plotted over each data set. Legends for developmental changes that were statistically significant are indicated in green font while features that were developmentally stable are indicated in red.

Examples of the physiologic responses of putative HVC_RA_ neurons can be seen in Figure 5. A total of *n =* 35 putative HVC_RA_ neurons were recorded across development: *n =* 8 at subsong, *n =* 11 at plastic song, and *n =* 16 at adult song. The HVC_RA_ cells shown are termed “putative” although we are confident in our classification based on the comparative similarity to previously identified adult physiology, the lack of fluorescent labeling, and dissimilarity to the characteristic physiology of interneurons and identified HVC_X_ neurons. Figure 6 shows all data points for some of the key features that were measured in HVC_RA_ neurons. Developmental changes in properties of HVC_RA_ neurons that reached statistical significance included a decrease in resting potential and an increase in spike amplitude (Fig. 6, green headings). No large or consistent changes were observed in the HVC_RA_ membrane time constant, membrane input resistance, membrane capacitance, V_drop_, SR, rebound depolarization, spike frequency, adaptation rate, or spike width.

#### Passive cellular properties

The passive membrane properties of HVC_RA_ neurons are largely stable except for a decrease in the membrane resting potential over development (Table 1). The resting membrane potential at subsong averaged −61 ± 2.05 mV, decreasing to an average of −66 ± 0.94 mV at plastic song, before reaching −77 ± 0.57 mV in adults. The resting potential observed in adult HVC_RA_ neurons is comparable with values previously reported (Dutar et al., 1998; Mooney and Prather, 2005; Daou et al., 2013). The membrane time constant, input resistance, and membrane capacitance were largely stable across development. However, subsong HVC_RA_ neurons trended toward a higher membrane time constant averaging 28 ± 4.09 ms, decreasing at plastic song, averaging 17 ± 2.48 ms, before reaching an average of 18 ± 2.53 ms at adult song. The difference in the membrane time constant across developmental timepoints, however, was not statistically significant.

#### Response to hyperpolarizing applied currents

The measurement of V_drop_ during hyperpolarization was stable across development, averaging 30 ± 6.55 mV at subsong, 29 ± 6.01 at plastic song and 28 ± 3.8 mV at adult song. Unlike HVC_X_ neurons, HVC_RA_ neurons show no sag in response to hyperpolarizing currents across development and they show only minor rebound depolarization.

#### Response to depolarizing applied currents

Adult HVC_RA_ neurons fired at lower frequencies than HVC_X_ and HVC_INT_ neurons, and often fired a single or just a few action potentials. There was an increase in the spike amplitude across development beginning at an average amplitude of 50 ± 6.39 mV during subsong, increasing to 64 ± 3.29 mV at plastic song, reaching 77 ± 3.59 mV at adult song. There was no significant change in spike width across development. The HVC_RA_ neurons shown in Figure 5 all fired at the onset of the current injection; however, a subset of HVC_RA_ neurons exhibited a spike delay. These neurons typically fired at higher frequencies than their onset-spiking counterparts. Both phenotypes have previously been reported in adults (Daou et al., 2013), and were observed at all behavioral timepoints studied.

#### Mathematical models suggest developmental changes in channel parameters of HVC_RA_ neurons

Fitting the model to data from Figure 5*A* suggests that the developmental change in resting membrane potential of the HVC_RA_ neurons is largely due to the leak current. The adult HVC_RA_ models showed a less negative reversal potential and an increase in the conductance of the leak current relative to earlier experimental timepoints (Table 2).

While HVC_RA_ neurons show no sag in response to hyperpolarizing currents, there is variation in V_drop_ across cells. The subsong trace shown in Figure 5 has a greater V_drop_ when compared to the other two examples. As a population, however, there was no age-related change in V_drop_ (Table 1). Nevertheless, the model results suggest that variation in V_drop_ among HVC_RA_ neurons could be explained by differences in the fast component of the h-current. As such, the h-current may be playing a significant role in shaping HVC_RA_ physiology despite the lack of a sag response, which reflects the slow component of the h-current.

**Figure 7.**
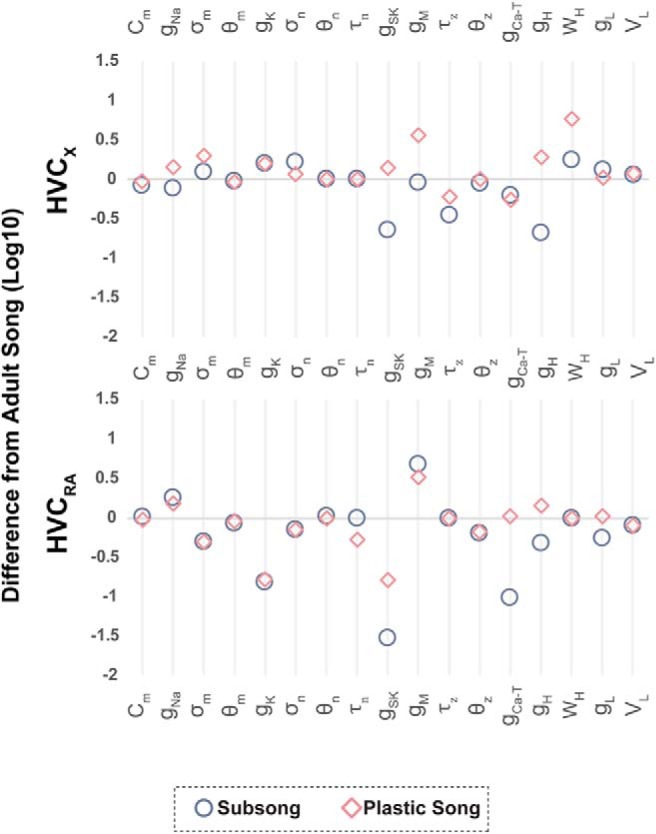
Developmental variation in ion channel parameters relative to adult. Model parameters that differed from the adult cell model are listed along the *x*-axis. Parameters that did not change are not displayed but can be found in Table 2. All parameter values were manually fit to representative data from Figures 3, 5. In this plot, conductance (*g*) values were first normalized to the capacitance of the model neuron. All values are displayed as a ratio comparing the juvenile model parameters to the adult (on a logarithmic scale). A value of zero indicates no difference relative to the adult. A value >0 indicates an increase in the magnitude of the parameter, and a value <0 indicates a decrease in the magnitude of the parameter relative to the adult. The models predict that HVC_X_ and HVC_RA_ neurons undergo different changes in ionic conductances as a function of development.

As in HVC_X_ neurons, spike amplitude was largely set by *g_Na_* and *g_K_*. The experimental results show that adult HVC_RA_ neurons have a larger spike amplitude than juveniles. Counterintuitively, Figure 7 shows *g_Na_* to be smaller in adult neurons than in the juvenile neurons. This can be explained by the concurrent increase in *g_K_* in adults, which regulates the spike after-hyperpolarization. The SK current plays a large role in limiting the number of spikes produced during the depolarization pulse in HVC_RA_ neurons. This is consistent with Daou et al. (2013), their Figure 12, which showed a single spiking HVC_RA_ neuron exhibiting tonic firing following the application of an SK channel blocker, apamin. Additionally, the SK current along with the M-current sets the voltage plateau, the steady state voltage following the spike(s). The model suggests that there is a developmental increase in the SK conductance (Table 2; Fig. 7); however, at all timepoints *g_SK_* is large enough to terminate the spiking during a 200 pA current pulse. Figure 7 shows that the juvenile models have a larger *g_M_* when compared to the adults. The models suggest that the larger conductance coincides with differences in the kinetics of the M-current, such that the activation curve of juveniles is shifted rightwards, activating at higher voltages, compared to the adults (Fig. 7, negative value of the θ_*z*_ comparison). This type of developmental change in kinetics is consistent with other model systems (Protas et al., 2003).

### Interneurons show more stability over development

The intrinsic physiology of HVC_INT_ neurons was more stable over development than in HVC_X_ or HVC_RA_ neurons. A total of *n =* 25 HVC_INT_ neurons were recorded across development: *n =* 6 at subsong, *n =* 10 at plastic song, and *n =* 9 at adult song. The classification of HVC interneurons was based on the similarity in their physiology to that of adults, their lack of labeling, and dissimilarity to HVC_RA_ and HVC_X_ neurons. No dramatic developmental changes were identified in any of the features measured in the putative interneurons. This indicates that the physiologic changes identified in HVC_X_ and HVC_RA_ neurons are not global changes across HVC, but cell-type-specific changes.

#### Passive membrane properties

The spontaneous spiking of HVC_INT_ neurons made it difficult to reliably measure passive membrane properties, so these were not analyzed. Instead we focused on the active properties of these neurons. Note that HVC_INT_ neurons display spontaneous firing although all experiments were conducted in the presence of synaptic blockers. This phenotype was observed throughout development.

#### Response to hyperpolarizing applied currents

**Figure 8.**
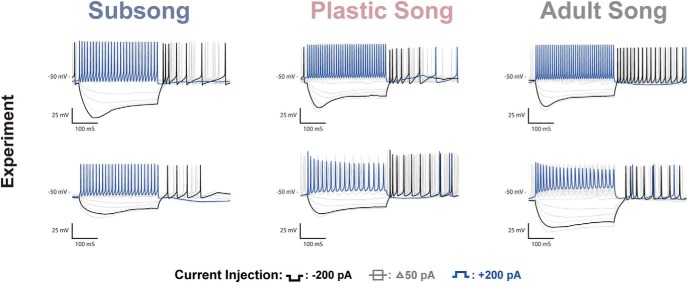
HVC_INT_ neurons are comparatively stable across development. Voltage traces of HVC_INT_ neurons recorded at subsong, plastic song, or adult stages of development. As a population, HVC_INT_ neurons show variability in many of the measured features; however, the distributions of these measured features were relatively stable across developmental timepoints (Table 1). Cells from all three age groups show high firing frequencies, a large sag and rebound firing. The cells shown here provide examples of the variability observed in HCV_INT_ neurons, however, there was no consistent classifiable difference across groups for any of the features analyzed.

HVC_INT_ neurons showed a comparable V_drop_ during hyperpolarization with values largely overlapping at each developmental time point. They exhibited large inward rectifying responses to hyperpolarizing currents that were observed throughout development. Interneurons averaged a SR of 0.13 ± 0.0267 at subsong, 0.11 ± 0.0190 at plastic song, and 0.13 ± 0.0204 at adult song. At all developmental timepoints, this ratio was much larger than that observed in HVC_X_ neurons, resulting in a well-defined “sag” in the voltage (Fig. 8). Similar to adult HVC_X_ neurons, HVC_INT_ neurons fired a rebound spike or burst of spikes following hyperpolarization. However, unlike HVC_X_ neurons, HVC_INT_ neurons exhibited this phenotype across development (Fig. 8).

#### Depolarizing response

HVC_INT_ neurons fired at the highest frequencies of the three cell types, with very little spike frequency adaptation. HVC interneurons had similar spike amplitudes across development, averaging 67 ± 6.00 mV during subsong, 64 ± 3.02 mV at plastic song, and 58 ± 2.17 mV at adult song, with large overlap in the three developmental timepoints. Spike width was similarly stable, averaging 0.94 ± 0.16 ms during subsong, 1.44 ± 0.18 ms during plastic song, and 1.10 ± 0.13 ms during adult song.

## Discussion

We characterized the intrinsic physiology of HVC neurons as a function of vocal development and identified population-specific changes in the intrinsic physiology of HVC projection neurons. Additionally, we modeled the intrinsic physiology of HVC neurons at each developmental time point, implicating specific changes in the pattern of ion channel expression as a function of vocal development. Given the established role of Area X in song learning (Sohrabji et al., 1990), the developmental change in the response of HVC_X_ neurons to hyperpolarizing currents was particularly striking. Indeed, juvenile HVC_X_ neurons lacked many of the phenotypic features held by adult HVC_X_ neurons. At subsong they showed no sag response and no rebound spiking or large after-depolarization. By plastic song, HVC_X_ neurons showed an increased variation in the magnitude of their sag response such that some neurons show little to no sag response and some showed adult-like levels. Still, we did not observe rebound spiking in plastic HVC_X_ neurons, but did see a moderate after-depolarization. Adult HVC_X_ neurons showed an increase in sag and V_drop_ compared to juveniles and often exhibited rebound firing or a comparatively large after-depolarization. HVC_RA_ neurons also changed over development but in different ways than HVC_X_ neurons. There was a downward shift in the HVC_RA_ cell’s resting membrane potential over development and an increased spike amplitude. In general, the intrinsic physiology of HVC interneurons appeared much more stable across development, maintaining many of the phenotypic properties observed in adults. Overall, we determined that the intrinsic physiology of HVC projection neurons, but not interneurons, changes across vocal development.

### Relationship to prior findings

To our knowledge this is the first systematic exploration of the intrinsic physiology of HVC neurons over vocal development. Our results complement the work of others characterizing the physiology of HVC under other conditions (Dutar et al., 1998; Schmidt and Perkel, 1998; Mooney and Prather, 2005; Long et al., 2010; Roberts et al., 2010; Peng et al., 2012; Stauffer et al., 2012). This project also helps to clarify some of the data presented in Kubota and Taniguchi (1998), one of the earliest attempts to detail the intrinsic physiology of HVC neurons. Their study recorded from juveniles from 37 to 54 dph; however, the specific ages at which each recording was taken were not reported. While Kubota and Taniguchi (1998)’s characterization of HVC neurons influenced our modeling work, we noticed discrepancies between their recordings and those that we reported in Daou et al. (2013), which were taken in adults. The results presented here (Figs. 3–7) suggest that the discrepancies were due to developmental differences in intrinsic physiology. A notable example is the rebound firing in HVC_X_ neurons observed in Daou et al. (2013), but not reported by Kubota and Taniguchi (1998). Their recordings however, show similarities to what we report here in juveniles. Additionally, the subsong HVC_RA_ neurons reported here show similarities to what Kubota and Taniguchi (1998) classified as “type 4” cells, which exhibited a resting potential near −70 mv and a “slower” hyperpolarizing response. They hypothesized that these were “immature” HVC_RA_ cells. The present data suggest their hypothesis was accurate. Given these differences it is important to recognize that the intrinsic physiology of juvenile and adult neurons is not always equivalent.

### Identifying sources of developmental stability and plasticity

Among the three classes of HVC neurons, HVC_X_ and HVC_RA_ neurons show the most dramatic disparity in the stability of their intrinsic physiology across development. HVC_X_ neurons show a pronounced developmental shift in the response to inhibitory currents while HVC interneurons, the source of those inhibitory currents, remain largely stable throughout development. It has been shown that inhibition in HVC plays an important role in both song production (Kosche et al., 2015) and song learning (Vallentin et al., 2016). Vallentin et al. (2016) showed evidence that inhibitory activity during tutor song playback increased for previously learned syllables, suggesting that inhibition is playing a stabilizing role during learning. These results suggest that as the juvenile is learning to sing, synaptic changes lead to an increase in coordinated inhibitory activity. It is possible that the change in inhibitory response of HVC_X_ neurons, in conjunction with this increased inhibitory coordination is regulating song learning and stability. One caveat is that our classification criteria for interneurons is based solely on their physiologic profile, as retrograde labeling of interneurons is not possible. As such, a putative juvenile interneuron was classified as an interneuron given its similarity to the known physiology of the adult. This would naturally bias the results toward the conclusion that there is little change over development. Nevertheless, it is noteworthy that one can readily find adult-like interneurons in the juvenile HVC, which cannot be said for the projection neurons. If the intrinsic physiology of HVC interneurons is in fact established before onset of subsong, as our data suggest, then developmental changes in intrinsic interneuron physiology are not a component of, nor a requirement for, song learning. This leaves either change in the physiology of HVC projection neurons and/or changes in connectivity between all classes of HVC neurons as being the key components related to song learning.

From a theoretical perspective, it is commonly assumed that changes in synaptic connectivity carry the bulk of any learning-induced change in neural function and, in fact, there is evidence that such changes occur in HVC (Roberts et al., 2010, 2012; Day et al., 2013). There is also evidence that there are developmental changes in synaptic connectivity in RA (Wang and Hessler, 2006; Shank and Margoliash, 2009). It seems equally common to assume that the intrinsic properties of the component neurons are stable during learning and do not contribute to the ultimate changes in the input-output function of the neural circuit. The age-related changes in physiology observed in the present study challenge this latter assumption. It is possible that changes in the physiology of the component neurons, not just the synaptic strengths of their connections, contributes to song learning. Alternatively, or in parallel, the developmental changes in intrinsic physiology could interact with mechanisms of synaptic plasticity. For example, perhaps the developmental changes in physiology provide a means to regulate when the component neurons are most sensitive to conditions that can produce synaptic plasticity. Research by Sim et al. (2013) has shown that artificially increasing the intrinsic excitability of individual neurons in the Dentate Gyrus leads to synaptic changes. Additionally, they showed that changes in input connectivity are regulated by the activity-dependent transcription factor Npas4. It remains to be seen if the same molecular factors regulate synaptic plasticity in HVC; however, it does illustrate that changes in intrinsic physiology can alter the connectivity of a circuit. Given this, it is possible that the more depolarized resting potential of juvenile HVC_RA_ neurons makes them more prone to synaptic plasticity, and the later developmental changes in channels that lead to a more hyperpolarized resting potential make them resistant to further change. Such a mechanism could provide an underlying mechanism for closing the developmental sensitive period for song learning.

**Figure 9.**
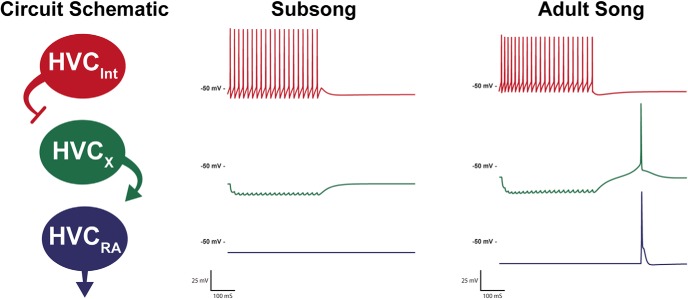
Developmental changes in channel properties can affect output of a simple circuit. The schematic depicts a simple circuit model in which the HVC_INT_ neurons are stimulated, inhibiting HVC_X_ neurons that in turn excite HVC_RA_ neurons. The subsong and adult song model circuits vary only in their intrinsic physiology, maintaining the same synaptic weighting between neurons. The subsong circuit reflects the physiology of the neurons recorded during subsong, and the adult song circuit reflects the physiology of the neurons recorded during adult song. The circuit is not necessarily indicative of all microcircuitry within HVC, but rather, to show that the developmental changes in HVC intrinsic physiology observed in the present experiments are of sufficient magnitude to have a significant influence on the output of even a very simple, but plausible, HVC local circuit.

At present, we cannot determine if the changes in physiology are driven by song learning, or if they set the developmental window for learning, but we can test the hypothesis that the magnitude of the changes in intrinsic physiology observed in the present experiments are adequate to change the output of a neural circuit. To illustrate the dramatic effect that alterations in intrinsic physiology can have on the output of a circuit, we used the model neurons developed in this study to create a simple circuit model (Fig. 9), one based on an HVC network model presented in Bertram et al. (2014). The circuit consists of an interneuron synaptically connected to an HVC_X_ neuron which is in turn connected to an HVC_RA_ neuron. This pattern of connectivity is consistent with the known synaptic connectivity within HVC (Mooney and Prather, 2005), although more complex patterns of connectivity are certainly possible (Bertram et al., 2014; Kosche et al., 2015; Kornfeld et al., 2017). We present two circuits, one with the intrinsic physiology of subsong neurons and one with the intrinsic physiology of the adult neurons. Both circuits maintain the exact same pattern of connectivity and synaptic weighting. Despite these circuits having the same connectivity, the output varies greatly based solely on differences in the intrinsic physiology of the constituent neurons. In the subsong circuit the inhibitory input to the HVC_X_ neuron does not elicit rebound firing and, as such, cannot activate the subsong HVC_RA_ neuron to drive singing. Meanwhile, in the adult circuit the inhibitory input to the HVC_X_ neuron from the interneuron triggers rebound firing which in turn activates the HVC_RA_ neuron. Lack of an output response in the subsong circuit is consistent with the findings of Aronov et al. (2008), where it was shown that HVC_RA_ neurons make little to no premotor contribution to subsong, which is driven instead by lateral portion of the magnocellular nucleus of the anterior nidopallium (LMAN)_RA_ neurons.

While our results and simulations do not show that learning is a result of nonsynaptic plasticity in the biophysical properties of neurons, they do suggest that such plasticity can drive significant changes in the input-output function of a neural circuit. The possibility that component neurons of a circuit might change should be more widely considered in analyses of learning and memory (Debanne et al., 2003; Mozzachiodi and Byrne, 2010; Sehgal et al., 2013). To understand how the brain encodes a behavioral pattern it is important to know the intrinsic physiology of the constituent neurons along with their pattern of connectivity. The process of learning may involve modification of either (or both) of these properties.

## Synthesis

Reviewing Editor: Darcy Kelley, Columbia University Decisions are customarily a result of the Reviewing Editor and the peer reviewers coming together and discussing their recommendations until a consensus is reached. When revisions are invited, a fact-based synthesis statement explaining their decision and outlining what is needed to prepare a revision will be listed below. Note: If this manuscript was transferred from JNeurosci and a decision was made to accept the manuscript without peer review, a brief statement to this effect will instead be what is listed below.

I have reviewed the revised manuscript and find that it effectively addresses the concerns of the previous reviewers.
